# SARS-CoV-2 testing uptake and its determinants in six ethnic groups living in Amsterdam, the Netherlands: a registry-based study within the HELIUS cohort

**DOI:** 10.1093/eurpub/ckag081

**Published:** 2026-07-08

**Authors:** Sophie L Campman, Anders Boyd, Nina van Wilgen, Felix P Chilunga, Liza Coyer, Janke Schinkel, Charles Agyemang, Henrike Galenkamp, Anitra D M Koopman, Jelle Koopsen, Matthijs Welkers, Karien Stronks, Maria Prins

**Affiliations:** Department of Infectious Diseases, Public Health Service of Amsterdam, Amsterdam, The Netherlands; Department of Infectious Diseases, Amsterdam UMC location University of Amsterdam, Amsterdam, The Netherlands; Amsterdam Institute for Immunology and Infectious Diseases, Amsterdam, The Netherlands; Department of Infectious Diseases, Public Health Service of Amsterdam, Amsterdam, The Netherlands; Department of Infectious Diseases, Amsterdam UMC location University of Amsterdam, Amsterdam, The Netherlands; Amsterdam Institute for Immunology and Infectious Diseases, Amsterdam, The Netherlands; Stichting hiv monitoring, Amsterdam, The Netherlands; Department of Infectious Diseases, Public Health Service of Amsterdam, Amsterdam, The Netherlands; Department of Public and Occupational Health, Amsterdam UMC location University of Amsterdam, Amsterdam, The Netherlands; Amsterdam Public Health, Health Behaviors and Chronic Diseases, Amsterdam, The Netherlands; Department of Infectious Diseases, Public Health Service of Amsterdam, Amsterdam, The Netherlands; Department of Infectious Diseases, Amsterdam UMC location University of Amsterdam, Amsterdam, The Netherlands; Amsterdam Institute for Immunology and Infectious Diseases, Amsterdam, The Netherlands; Department of Medical Microbiology and Infection Prevention, Amsterdam UMC location University of Amsterdam, Amsterdam, The Netherlands; Department of Public and Occupational Health, Amsterdam UMC location University of Amsterdam, Amsterdam, The Netherlands; Division of Endocrinology, Diabetes and Metabolism, Department of Medicine, Johns Hopkins University School of Medicine, Baltimore, MD, United States; Department of Public and Occupational Health, Amsterdam UMC location University of Amsterdam, Amsterdam, The Netherlands; Amsterdam Public Health, Health Behaviors and Chronic Diseases, Amsterdam, The Netherlands; Department of Public and Occupational Health, Amsterdam UMC location University of Amsterdam, Amsterdam, The Netherlands; Amsterdam Public Health, Health Behaviors and Chronic Diseases, Amsterdam, The Netherlands; Department of Infectious Diseases, Public Health Service of Amsterdam, Amsterdam, The Netherlands; Department of Infectious Diseases, Public Health Service of Amsterdam, Amsterdam, The Netherlands; Department of Public and Occupational Health, Amsterdam UMC location University of Amsterdam, Amsterdam, The Netherlands; Amsterdam Public Health, Health Behaviors and Chronic Diseases, Amsterdam, The Netherlands; Department of Infectious Diseases, Public Health Service of Amsterdam, Amsterdam, The Netherlands; Department of Infectious Diseases, Amsterdam UMC location University of Amsterdam, Amsterdam, The Netherlands; Amsterdam Institute for Immunology and Infectious Diseases, Amsterdam, The Netherlands

**Keywords:** SARS-CoV-2, COVID-19, PCR, testing uptake, ethnicity

## Abstract

Diagnostic testing was key in preventing transmission of severe acute respiratory syndrome coronavirus 2 (SARS-CoV-2) in the pre-vaccination era of the coronavirus disease 2019 pandemic. This study compared SARS-CoV-2 polymerase chain reaction (PCR) testing uptake and its determinants across six ethnic groups in Amsterdam, the Netherlands. We analyzed data from the population-based Healthy Life in an Urban Setting cohort linked to SARS-CoV-2 testing registry data from the Public Health Service of Amsterdam. Testing uptake was defined as completing at least one SARS-CoV-2 PCR test before 6 September 2021. We examined the association between ethnicity and testing uptake, and assessed determinants of testing uptake per ethnic group, using logistic regression while correcting for ethnic-specific age and sex distributions in Amsterdam. We included 19 006 participants (median age 53 years; 57% female). Testing uptake ranged from 25.3% (95% confidence interval (CI) = 23.1%–27.5%) in the Ghanaian to 52.2% (95% CI = 50.3%–54.1%) in the Turkish group. Individuals of Turkish origin [adjusted odds ratio (aOR) = 1.12, 95% CI = 1.01–1.23] were more likely, while those of African Surinamese (aOR = 0.88, 95% CI = 0.79–0.97) and particularly Ghanaian (aOR = 0.35, 95% CI = 0.30–0.40) origin less likely to be tested compared to those of Dutch origin. Younger age and perceived work or home-related stress were associated with testing uptake across most ethnic groups. Other determinants were specific to certain groups. SARS-CoV-2 testing uptake varied slightly across most ethnic groups in Amsterdam with the highest uptake among individuals of Turkish origin but was remarkably lower among individuals of Ghanaian origin. Given the diversity of identified determinants, testing strategies should be tailored to the needs of specific groups.

## Introduction

In the early phases of the coronavirus disease 2019 (COVID-19) pandemic, many countries implemented large-scale diagnostic testing for rapid identification of individuals infected with severe acute respiratory syndrome coronavirus 2 (SARS-CoV-2) [1]. Testing, combined with contact tracing, isolation of infected individuals and quarantine of their contacts, was the cornerstone in limiting SARS-CoV-2 transmission before vaccination became available [1, 2]. In response to widespread community transmission with limited testing capacity, the World Health Organization (WHO) initially recommended prioritizing testing for individuals at high risk of severe disease and healthcare workers [1]. As testing capacity increased, the WHO recommended expanding testing to contacts of individuals who tested positive for SARS-CoV-2, even if they had mild or no symptoms [3].

In the Netherlands, the SARS-CoV-2 testing policy was generally in line with WHO recommendations, and evolved over time when capacity and availability of testing sites (e.g. more locations, mobile testing units, walk-in options) increased [4]. SARS-CoV-2 testing was mostly conducted free-of-charge by testing centers affiliated with the Public Health Services (PHS) using a Nucleic Acid Amplification Test (NAAT)-based assay [generally polymerase chain reaction (PCR)]. As testing was voluntary, its effectiveness in limiting transmission largely depended on individual testing behavior. In a Dutch national survey conducted between November 2020 and April 2021, 51% of the participants with COVID-19-related symptoms reported they had been tested, with lower uptake observed among individuals over 65 years and those with lower educational levels [5].

Ethnic minority groups have experienced higher rates of infection, hospitalization, and mortality from SARS-CoV-2 [6], also in the Netherlands [7–9]. Early detection of infection could help mitigate severe disease progression, allowing individuals to seek timely medical care, and, together with isolation and quarantine, could reduce transmission within their social environments [10]. Studies have reported mixed findings with respect to SARS-CoV-2 testing uptake among those with a migration background, where studies in Spain, Italy and the USA found no differences, while a study conducted in the UK found lower testing proportions in individuals with an ethnic minority background [11–13]. No data on ethnic differences in testing are available in the Netherlands, and specifically in Amsterdam, where 60% of the population has a history of migration [14]. Understanding SARS-CoV-2 testing uptake across ethnic groups can help identify potential disparities, inform equitable testing strategies not only for COVID-19 but also for other diseases, and guide the preparation of large-scale testing strategies for future outbreaks of respiratory infectious diseases.

This study aimed to compare the SARS-CoV-2 testing uptake, conducted by the PHS using PCR, in individuals residing in Amsterdam, the Netherlands, of Dutch, South-Asian Surinamese, African Surinamese, Ghanaian, Turkish, and Moroccan ethnic origin. We also explored the reasons for differences in testing uptake with respect to demographic, social and cultural factors by undertaking a determinant analysis for each ethnic group.

## Methods

### Study design and population

We used data from the Healthy Life in an Urban Setting (HELIUS) study, which is a population-based multi-ethnic prospective cohort study conducted in Amsterdam that focuses on the causes of potential ethnic disparities in cardiovascular disease (CVD), mental health, and infectious diseases. Detailed procedures have been previously described [15]. Briefly, the HELIUS cohort comprises 24 781 adult individuals of Dutch, Surinamese, Ghanaian, Turkish, and Moroccan origin living in Amsterdam who were included between January 2011 and December 2015.

Individuals were randomly sampled, stratified by ethnic origin, through the municipality register of Amsterdam, and invited to participate. This register contains data on country of birth of residents and their parents, which we used to determine ethnic origin [16]. Ethnicity is a complex and largely socially constructed concept that encompasses multiple dimensions, such as country of birth, language, religion, and culture. There might therefore be some heterogeneity within ethnic groups. However, country of birth is a widely accepted and stable indicator for ethnic origin in the Netherlands, and Dutch studies have shown high correlation between country of birth and self-identified ethnicity among Turkish, Moroccan and Surinamese groups [16]. We defined ethnic origin groups other than Dutch as: (1) the individual and at least one parent were not born in the Netherlands (first-generation migrants), and (2) the individual was born in the Netherlands, but both parents were not (migrants’ offspring). Given the ethnic heterogeneity of the Surinamese population, we further classified participants with a Surinamese background into African, South-Asian, Javanese or “other” based on self-report during the baseline questionnaire [15, 16].

Participants completed a questionnaire and underwent physical examination during which biological samples were obtained. For this study, we excluded participants who did not complete both the physical examination and questionnaire at the HELIUS baseline visit. We also excluded individuals of unknown ethnic origin, and individuals of Javanese Surinamese or other Surinamese origin due to smaller sample sizes compared to the other ethnic groups.

The HELIUS study was approved by the Academic Medical Center Ethical Review Board, and written informed consent was obtained from all participants [15].

### Data linkage

HELIUS data were linked to SARS-CoV-2 PCR testing data registered by the PHS of Amsterdam in a database named CoronIT. Records dating between 29 April 2020 and 6 September 2021 were included [17]. These data did not include diagnostic SARS-CoV-2 tests conducted at other PHS locations in the Netherlands, commercial testing facilities (e.g. for international travel), general practitioners (GP), hospitals, employers (e.g. for healthcare workers), or at-home antigen self-tests. More detailed information on data linkage is presented in Supplementary Methods S1. Only HELIUS participants who had given permission for linkage to health registries and were alive on 1 March 2020 (i.e. the start of the SARS-CoV-2 pandemic) were eligible for linkage.

### Study outcome

SARS-CoV-2 testing uptake was defined as having at least one PCR test completed at a testing center affiliated with the PHS of Amsterdam by 6 September 2021, with both the test outcome (i.e. successfully completed) and its date recorded in CoronIT. We assumed that all participants who were eligible for linkage but were not linked to CoronIT data had not been tested for SARS-CoV-2.

### Study covariates

We selected several potential determinants of SARS-CoV-2 testing uptake, with a focus on factors related to pathways that may contribute to ethnic differences in SARS-CoV-2 exposure, susceptibility to infection, and disease severity [18]. We used the following data from the HELIUS baseline visit (2011–15): age (recalculated for age on 1 January 2021), sex, educational level (no school, elementary school, lower/intermediate vocational school or lower/intermediate secondary school vs. higher vocational or university education), household size, Dutch language proficiency, health literacy, cultural orientation [no integration (including separation and marginalization) vs. integration (including integration and assimilation)], perceived discrimination, experiencing stress at work or at home, and social support. Comorbidities were based on self-report, medication use or both: diabetes mellitus, asthma/Chronic Obstructive Pulmonary Disease (COPD), and CVD. Body mass index (BMI) was measured during physical examination at the baseline visit and categorized into underweight/healthy weight (BMI < 25 kg/m^2^), overweight (BMI 25–30 kg/m^2^), and obesity (BMI ≥ 30 kg/m^2^). Detailed information on the instruments used has been previously described [17, 19].

### Statistical analysis

The percentage of SARS-CoV-2 testing uptake was calculated per ethnic group and compared between ethnic groups using Pearson’s *χ*^2^ test. We explored changes in testing uptake across the four phases of testing policies, as described in Supplementary Methods S2.

We modeled the probability of testing uptake in different ethnic groups using logistic regression. We calculated the univariable odds ratio (OR) and its 95% confidence interval (CI) comparing the odds of being tested vs. not being tested across ethnic groups. We then examined the determinants of SARS-CoV-2 testing uptake within each ethnic group, separately. We chose to stratify these analyses *a priori* by ethnicity because we hypothesized that differences in determinants of testing uptake would exist between groups, as demonstrated for SARS-CoV-2 vaccination intent [19]. Ethnic-specific potential determinants (i.e. cultural orientation and Dutch language proficiency) were only included in the models for the other than Dutch ethnic groups. We constructed a multivariable model by including all potential determinants with a *P* values ≤ .20 in univariable analyses in a full model. After assessing covariate distributions and collinearity, we removed all determinants with an overall *P* values ≥ .05 in backward stepwise fashion to obtain a final model. To confirm whether certain factors were ethnic-specific, we tested interactions between ethnic group and determinants that remained in at least one final model. Further details of these analyses are provided in Supplementary Methods S3.

A *P* values <.05 was considered statistically significant. The percentage of SARS-CoV-2 testing uptake and regression models were corrected for the population structure of Amsterdam using post-stratification weights based on the age and sex distribution within each ethnic group in Amsterdam. All analyses were performed using STATA version 17.0 (College Station, TX, USA).

## Results

### Description of the study population

Of the 24 781 HELIUS baseline participants, we excluded those who had not provided informed consent for data linkage to health registries (*n *= 4180) or deceased before 1 March 2020 (*n *= 253). As such, 20 348 participants (82.1%) were eligible for linkage with registry data. We then excluded individuals who did not participate in the baseline physical examination or questionnaire (*n *= 847), or were of Javanese Surinamese (*n *= 217), other Surinamese (*n *= 235), or unknown (*n *= 43) ethnic origin. We analyzed the data of 19 006 participants (Supplementary Fig. S1).

Characteristics of participants included in the analysis are presented in Supplementary Table S1. Median age was 53 years [interquartile range = 41–62] and the majority was female (56.9%). The percentage of participants who attended higher vocational education or university ranged from 6.3% in the Ghanaian group to 60.3% in the Dutch group.

### Ethnic variation in SARS-CoV-2 testing uptake

Of all included participants, 45.1% had completed at least one PCR test for SARS-CoV-2. Participant characteristics differed slightly between those who did and did not test for SARS-CoV-2, with the exception of CVD, asthma or COPD, experiences of discrimination based on their background, and level of acculturation, for which no significant differences in testing were observed across levels of these characteristics (Supplementary Table S2).

**Figure 1.**
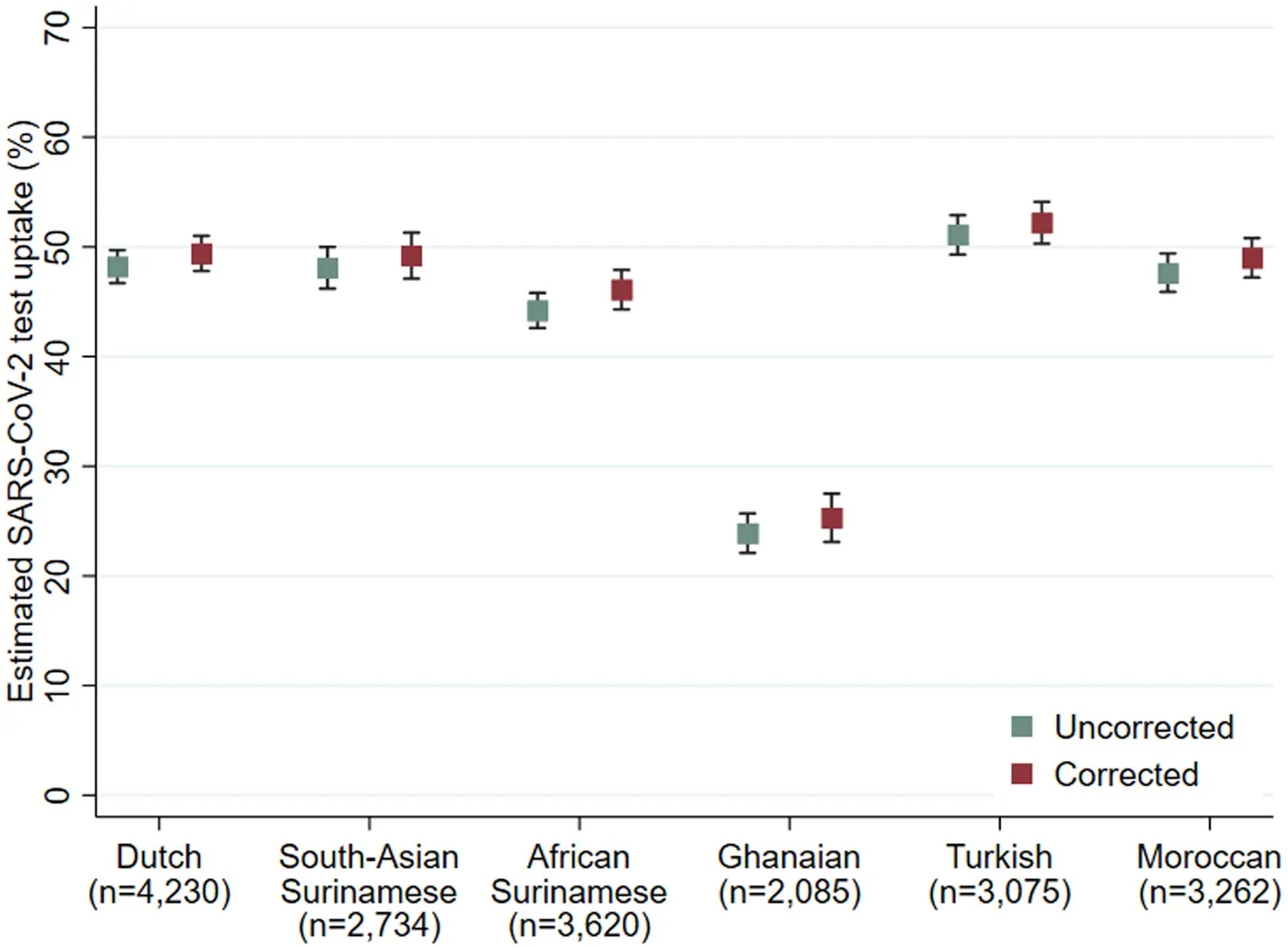
Uncorrected and corrected^a^ uptake of at least one SARS-CoV-2 test^b^ among HELIUS participants, as registered by the Public Health Service of Amsterdam, per ethnic group, Amsterdam, the Netherlands, 29 April 2020 to 6 September 2021 (*n* = 19 006). Boxes represent the estimated testing uptake, bands the corresponding 95% CI. ^a^Corrected uptake estimates account for the age and sex distribution of the Amsterdam population through post-stratification weights. ^b^PCR was used to detect SARS-CoV-2 and was conducted by a testing center affiliated with the Public Health Service of Amsterdam. Abbreviations: SARS-CoV-2, Severe Acute Respiratory Syndrome Coronavirus 2; HELIUS, Healthy Life in an Urban Setting; PCR, Polymerase chain reaction.

SARS-CoV-2 testing uptake differed between ethnic groups (Supplementary Table S1). The uncorrected and population-corrected testing uptake are shown across ethnic groups in Fig. 1. Individuals of Turkish origin had the highest testing uptake (corrected percentage 52.2%, 95% CI = 50.3%–54.1%), followed by the Dutch (49.4%, 95% CI = 47.8%–51.0%), South-Asian Surinamese (49.2%, 95% CI = 47.1%–51.3%), Moroccan (49.0%, 95% CI = 47.2%–50.8%), African Surinamese (46.1%, 95% CI = 47.1%–51.3%), and Ghanaian (25.3%, 95% CI = 23.1%–27.5%) groups. Among individuals with test uptake, 47.1% completed more than one test (ranging between 28.9% in the Ghanaian and 55.2% in the Dutch group) and 30.1% had at least one positive test result (varying between 15.0% in the Dutch and 42.5% in the Moroccan group) (Supplementary Table S1). Testing uptake fluctuated over time across ethnic groups (Supplementary Results S1, Supplementary Fig. S2). In most groups, testing uptake was highest during phase 2, when testing was available to all symptomatic individuals. Uptake in the Ghanaian group slightly increased across the four testing policy periods, a pattern not observed in the other groups.

**Figure 2.**
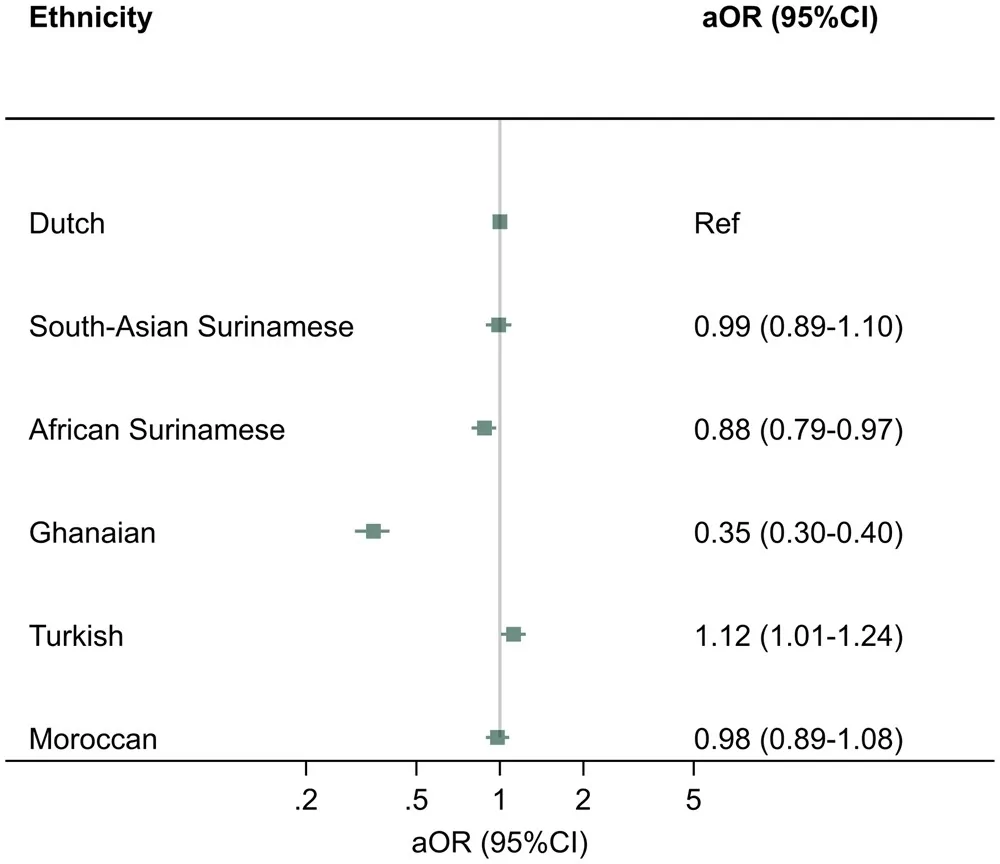
Ethnic variation in SARS-CoV-2 PCR testing uptake among HELIUS participants, Amsterdam, the Netherlands, 29 April 2020 to 6 September 2021 (*n* = 19 006). SARS-CoV-2 testing data was registered between 29 April 2020 and 6 September 2021 and was conducted by a testing center affiliated with the Public Health Service of Amsterdam. The association between ethnicity and the uptake of at least one SARS-CoV-2 test was examined using logistic regression. Model accounts for the age and sex distribution within ethnic groups of the Amsterdam population through post-stratification weights. SARS-CoV-2, Severe Acute Respiratory Syndrome Coronavirus 2; HELIUS, Healthy Life in an Urban Setting; PCR, Polymerase chain reaction; aOR, adjusted odds ratio; CI, confidence interval; Ref, Reference category.

In both uncorrected and population-corrected analyses, participants of African Surinamese (adjusted odds ratio [aOR] = 0.88, 95% CI = 0.79–0.97) and Ghanaian (aOR = 0.35, 95% CI = 0.30–0.40) origin were significantly less likely, while individuals of Turkish origin (aOR = 1.12, 95% CI = 1.01–1.23) more likely to test for SARS-CoV-2 at one of the testing centers affiliated with the PHS of Amsterdam compared to those of Dutch origin (Fig. 2, Supplementary Table S3).

### Ethnic-specific determinants of SARS-CoV-2 testing uptake

Univariable associations between potential determinants and SARS-CoV-2 testing uptake are presented for each ethnic group, separately, in Supplementary Tables S4–S9. In most final multivariable models (Table 1), individuals over 60 years were less likely to test than those under 30 years. In the South-Asian Surinamese and Moroccan groups, this association was also true for individuals aged 50–59 years. In the African Surinamese, Turkish, and Moroccan groups, females were more likely to test than males, and individuals who attended higher vocational or university education were more likely to test than those with other educational backgrounds. Individuals of Dutch, African Surinamese and Turkish origin living in larger households were more likely to get tested when compared to those living alone. Participants of Dutch, South-Asian Surinamese, African Surinamese, and Turkish origin who reported to ever experience stress at work or at home over the twelve months prior to the survey were more likely to test than those who never experienced stress.

**Table 1.** Determinants of SARS-CoV-2 PCR testing uptake per ethnic group (multivariable logistic regression analyses), Amsterdam, the Netherlands, 29 April 2020 to 6 September 2021

	Dutch (*n* = 4203)	South-Asian Surinamese (*n* = 2713)	African Surinamese (*n* = 3510)	Ghanaian (*n* = 2085)	Turkish (*n* = 2983)	Moroccan (*n* = 3224)	
**Variable**	aOR (95% CI)	aOR (95% CI)	aOR (95% CI)	aOR (95% CI)	aOR (95% CI)	aOR (95% CI)	
**Age in years** ^a^							
<30	Ref	Ref	Ref	Ref	Ref	Ref	
30–39	1.03 (0.76–1.41)	0.60 (0.42–0.87)	1.17 (0.80–1.71)	0.97 (0.59–1.62)	1.03 (0.78–1.38)	0.89 (0.68–1.16)	
40–49	0.93 (0.68–1.28)	0.69 (0.48–1.00)	1.12 (0.77–1.64)	0.68 (0.43–1.10)	1.05 (0.79–1.38)	0.87 (0.67–1.13)	
50–59	0.91 (0.67–1.24)	0.67 (0.47–0.94)	1.08 (0.76–1.53)	0.77 (0.50–1.18)	0.95 (0.74–1.24)	0.66 (0.50–0.86)	
60–69	0.83 (0.61–1.13)	0.52 (0.37–0.74)	0.79 (0.55–1.13)	0.51 (0.32–0.80)	0.51 (0.37–0.70)	0.54 (0.40–0.74)	
70–79	0.51 (0.37–0.72)	0.42 (0.28–0.63)	0.73 (0.49–1.10)	0.37 (0.14–0.97)	0.52 (0.29–0.90)	0.46 (0.28–0.73)	
**Female sex (vs. male)**	–	–	1.23 (1.05–1.43)	–	1.17 (1.00–1.37)	1.21 (1.04–1.41)	
**Higher vocational/university education (vs. other educational backgrounds)**	–	–	1.36 (1.14–1.64)	–	1.29 (1.02–1.63)	1.70 (1.37–2.12)	
**Number of people in household**							
1 (lives alone)	Ref	–	Ref	–	Ref	–	
2	0.92 (0.78–1.08)	–	1.19 (0.97–1.46)	–	1.57 (1.13–2.18)	–	
3	1.14 (0.92–1.40)	–	1.49 (1.20–1.85)	–	1.85 (1.24–2.54)	–	
4	1.28 (1.03–1.61)	–	1.34 (1.06–1.71)	–	2.06 (1.52–2.80)	–	
≥5	1.21 (0.84–1.74)	–	1.31 (0.97–1.77)	–	2.06 (1.51–2.81)	–	
**Asthma/COPD**	–	–	–	1.99 (1.07–3.70)	–	–	
**BMI**							
Underweight/healthy weight (BMI <25 kg/m^2^)	–	Ref	–	–	–	–	
Overweight (BMI 25–30 kg/m^2^)	–	1.30 (1.07–1.58)	–	–	–	–	
Obesity (BMI ≥30 kg/m^2^)	–	1.31 (1.03–1.66)	–	–	–	–	
**Low subjective health literacy (vs. adequate)**	–	–	–	–	–	0.76 (0.62–0.93)	
**Ever experienced stress at work or at home**	1.18 (1.01–1.39)	1.41 (1.17–1.70)	1.23 (1.05–1.45)	–	1.21 (1.02–1.44)	–	
**Limited social support (vs. sufficient)**	0.76 (0.65–0.90)	–	–	–	–	–	

Models account for the age and sex distribution of the Amsterdam population through post-stratification weights.

a Recalculated to age on January 1, 2021.

PCR, polymerase chain reaction; SARS-CoV-2, severe acute respiratory syndrome coronavirus 2; HELIUS, healthy life in an urban setting; aOR, adjusted odds ratio; CI, confidence interval; Ref, reference category; NA, not applicable; COPD, chronic obstructive pulmonary disease; BMI, body mass index.

Other determinants of testing uptake were having asthma or COPD (Ghanaian), having overweight or obesity (South-Asian Surinamese), reporting adequate health literacy (Moroccan), and experiencing sufficient social support (Dutch). More migration-related factors such as discrimination, cultural orientation, and Dutch language proficiency were not significantly associated with testing uptake. Results of the interaction analyses between ethnicity and determinants are presented in Supplementary Results S2.

## Discussion

This study, conducted in a large cohort in Amsterdam, the Netherlands, revealed slight differences in the uptake of SARS-CoV-2 PCR testing between adults from different ethnic backgrounds during the first three waves of infections [4], spanning from the rollout of testing by the PHS in early 2020 to widespread access to testing until 6 September 2021. Individuals of Turkish origin had slightly higher testing uptake than individuals of Dutch origin, while those of African Surinamese and particularly Ghanaian origin had lower testing uptake. The determinants of testing uptake varied across ethnic groups, although older age was consistently associated with lower testing uptake in all groups.

Testing uptake estimates, corrected for ethnic-specific age and sex distributions in Amsterdam, were comparable, ranging from 46% in the African Surinamese to 52% in the Turkish group, except for the Ghanaian group, with an uptake of 25%. This finding aligns with studies from Spain, Italy, and the USA, which reported similar uptake between residents with and without a migration background [11, 13]. The similar uptake across groups is noteworthy given the typically lower participation in SARS-CoV-2 vaccination program, the Dutch childhood National Immunization Program, and in cancer screening programs among people with a migration background, particularly among Turkish and Moroccan groups [17, 20, 21]. However, unlike childhood vaccination and cancer screening, SARS-CoV-2 testing was implemented during a pandemic and targeted the general population. Our findings on SARS-CoV-2 testing uptake might then not be directly applicable to these other contexts.

SARS-CoV-2 testing uptake was particularly low in the Ghanaian group. In contrast to the findings from Italy, Spain and the USA [11, 13], ethnic differences in SARS-CoV-2 testing uptake have previously been reported in the UK, with slightly lower uptake in South-Asian, Black, mixed and other (e.g. Chinese) ethnicities compared to White ethnicities between 1 September and 31 December 2020 [12]. Early pandemic data from a subsample of HELIUS participants demonstrated a high prevalence of SARS-CoV-2 infections in the Ghanaian group compared to the other ethnic groups studied [22]. However, they reported fewer symptoms, suggesting asymptomatic infections, lower symptom awareness, or reluctance toward disclosing symptoms, all potentially reducing testing. Individuals of Ghanaian origin expressed greater concern about disclosure and public attitudes toward SARS-CoV-2 infection than other groups, possibly contributing to experiences of stigma at testing sites [19]. Public health professionals similarly noted these fears during informal conversations with the community (COVID-19 prevention team, PHS of Amsterdam, personal communication). Testing uptake slightly increased over time in the Ghanaian group, possibly reflecting intensified PHS outreach efforts to raise COVID-19 awareness in the Ghanaian community through trusted figures, local radio, and churches. Additionally, practical barriers, such as limited testing availability when infection rates in the Ghanaian group were high, might have also played a role in testing uptake.

The proportion of individuals testing positive for SARS-CoV-2 was higher in the studied ethnic minority groups compared to the Dutch group. These higher PCR testing positivity rates in ethnic minority groups mirror findings from the UK, where individuals of South Asian, Black, mixed, and other ethnic backgrounds tested positive more frequently than individuals of White ethnic origin [12]. Our findings might reflect differences in testing patterns. Ethnic minority groups possibly tested mainly when symptomatic, potentially related to testing barriers such as difficulty recognizing symptoms, stigma, uncertainty about eligibility, access issues, and concerns about the consequences of a positive test result (e.g. income loss, job insecurity due to mandatory isolation protocols, and stigma) [23, 24]. Since we did not collect data on reasons for testing, we cannot infer why certain ethnic groups tested less.

We identified several common determinants of SARS-CoV-2 testing uptake across ethnic groups. Individuals over 60 years of age were less likely to be tested in most ethnic groups, which might be related to the lower risk of infection from smaller social networks and higher compliance with COVID-19 mitigation measures, such as physical distancing or visitor restrictions [25, 26]. On the other hand, older individuals might be less mobile, at higher risk for severe disease, may have been tested elsewhere (e.g. by their GP), and were thus missed in the PHS data. Females were more likely to test in the African Surinamese, Turkish, and Moroccan origin groups, aligning with a study that demonstrated higher compliance to COVID-19 government recommendations among females [26]. Individuals with higher vocational or university education, vs. those with other educational backgrounds, were more likely to be tested in the African Surinamese, Turkish, and Moroccan origin groups. The Dutch National Institute of Public Health and the Environment has reported similar associations between testing behavior and age, sex and educational level but did not examine whether these associations differed between ethnic groups [27]. Living in multi-person households was associated with higher testing uptake among Dutch, African Surinamese and Turkish individuals, possibly reflecting higher exposure risk through increased social contacts, as well as concerns about transmitting the virus to household members [28]. Lastly, previous experiences of stress at work or home were associated with higher testing uptake among individuals of Dutch, South-Asian Surinamese, African Surinamese, and Turkish origin. Although the exact reason is unclear, experiencing stress in general during the pandemic was generally associated with greater health concerns or working in essential occupation sectors (e.g. healthcare, education, public services) [29, 30], which likely contributed to increased testing.

Since other determinants were unique to specific ethnic groups, interventions aiming to increase testing would have to be tailored to specific groups rather than generalized. For example, not experiencing sufficient social support was associated with lower testing uptake in individuals of Dutch origin and lower health literacy with lower testing uptake among individuals of Moroccan origin. The latter could be addressed through providing educational campaigns, and simplified, accessible, culturally appropriate health information in the native language [31]. To foster trust between stakeholders, these programs would need to be carried out and information disseminated with community-based outreach programs or trusted community leaders. Whether such interventions are effective in increasing testing uptake should be further explored.

Our study has some limitations worth mentioning. First, individuals could have been misclassified as never testing. SARS-CoV-2 testing was registered in CoronIT from May 2020 onwards, with earlier tests recorded elsewhere and thus unavailable in our data. Additionally, our data did not include tests conducted at testing sites other than the PHS of Amsterdam. We examined whether testing at sites other than at the PHS differed between ethnic groups using data from a COVID-19 substudy conducted among a subsample of the HELIUS cohort between November 2020 and June 2021. These data revealed that 37% of individuals of Ghanaian origin reported being tested only outside the PHS sites (e.g. at work or at a commercial testing site), compared to 14%–22% in other groups (data not shown). These data suggest that uptake was likely more underestimated in the Ghanaian group than in other groups, although the uptake of SARS-CoV-2 testing in general as self-reported during the substudy visit, since their previous visit between June and October 2020, was the lowest in this group. Furthermore, our data (May 2020 to September 2021) did not cover the entire period of COVID-19 mitigation measures in the Netherlands (until March 2022). However, as self-testing became more common from March 2021 onwards, reducing the need for PCR testing through the PHS, our estimates would unlikely be biased. Furthermore, changes may have occurred in psychosocial, access-to-healthcare, cultural orientation, and comorbidity variables measured during the HELIUS baseline visit (2011–15) and the period of SARS-CoV-2 testing (2020–21). Additionally, some potential determinants related to testing uptake, such as testing center hours, travel time to testing site [32] or working in essential contact occupations [33], could not be addressed.

In conclusion, SARS-CoV-2 testing uptake at the PHS of Amsterdam was similar between individuals of Dutch, South-Asian Surinamese, and Moroccan origin in Amsterdam, but was much lower among individuals of Ghanaian origin, slightly lower among individuals of African Surinamese origin and somewhat higher among those of Turkish origin. It should be noted that ethnic groups are not homogenous. The diversity of some of the identified determinants across groups shows within-group variation in testing uptake. Subgroup heterogeneity should be further investigated, and interventions to increase testing uptake for disease control and future pandemic response should be flexible and inclusive to fit specific needs within groups as well.

## Supplementary Material

ckag081_Supplementary_Data

## Data Availability

The HELIUS data are owned by the Amsterdam University Medical Centers, location AMC in Amsterdam, The Netherlands. Any researcher can request the data by submitting a proposal to the HELIUS Executive Board as outlined at http://www.heliusstudy.nl/en/researchers/collaboration, by email: heliuscoordinator@amsterdamumc.nl. The HELIUS Executive Board will check proposals for compatibility with the general objectives, ethical approvals and informed consent forms of the HELIUS study. There are no other restrictions to obtaining the data and all data requests will be processed in the same manner. Key points This study examined SARS-CoV-2 testing uptake among six ethnic groups in Amsterdam. Testing uptake varied slightly across ethnic groups with the highest uptake in the Turkish group, except in the Ghanaian group, who had lower uptake. Older age was associated with lower testing uptake across all groups, while other determinants varied between groups. Tailored strategies are needed that account for the different determinants of testing across ethnic groups to strengthen testing campaigns for disease control and prepare for future pandemics.
