# Supplementary material for: SARS-CoV-2 testing uptake and its determinants in six ethnic groups living in Amsterdam, the Netherlands: a registry-based study within the HELIUS cohort
Source: Eur J Public Health. 2026 Jul 8;36(4):ckag081. doi: 10.1093/eurpub/ckag081 (PMC13344431; doi:10.1093/eurpub/ckag081)
Supplement: ckag081_Supplementary_Data [file ckag081_supplementary_data.docx]

**Supplementary materials to:**

**SARS-CoV-2 testing uptake and its determinants in six ethnic groups living in Amsterdam, the Netherlands: a registry-based study within the HELIUS cohort**

**Authors**

Sophie L. Campman, Anders Boyd, Nina van Wilgen, Felix P. Chilunga, Liza Coyer, Janke Schinkel, Charles Agyemang, Henrike Galenkamp, Anitra D.M. Koopman, Jelle Koopsen, Matthijs Welkers, Karien Stronks, Maria Prins.

**Contents**

**Methods S1**. Linkage of HELIUS data with SARS-CoV-2 testing and vaccination registry data.

**Methods S2**. Phases of SARS-CoV-2 testing policy in the Netherlands.

**Methods S3**. Interaction analyses between ethnicity and determinants of SARS-CoV-2 testing uptake.

**Results S1**. Variation in PCR testing uptake over time.

**Results S2**. Results of the interaction analyses between ethnicity and determinants of SARS-CoV-2 testing uptake.

**Figure S1.** Flowchart of the inclusion of HELIUS participants, Amsterdam, the Netherlands.

**Figure S2**. Percentage of HELIUS participants that had at least one successfully completed test appointment during different phases of the Dutch SARS-CoV-2 testing policy, Amsterdam, the Netherlands.

**Table S1. Characteristics of HELIUS participants who were eligible for linkage with registry data, per ethnic group, Amsterdam, the Netherlands**

**Table** **S2**. Characteristics of HELIUS participants for those who were and were not tested for SARS-CoV-2 (April 29, 2020 to September 6, 2021), Amsterdam, the Netherlands.

**Table S3**. Ethnic variation in SARS-CoV-2 testing uptake among HELIUS participants, Amsterdam, the Netherlands, April 29, 2020 to September 6, 2021 (n=19,006).

**Table S4**. Univariable and multivariable models with potential determinants of SARS-CoV-2 testing uptake among Dutch HELIUS participants, Amsterdam, the Netherlands, April 29, 2020 to September 6, 2021.

**Table S5**. Univariable and multivariable models with potential determinants of SARS-CoV-2 testing uptake among South-Asian Surinamese HELIUS participants, Amsterdam, the Netherlands, April 29, 2020 to September 6, 2021.

**Table S6**. Univariable and multivariable models with potential determinants of SARS-CoV-2 testing uptake among African Surinamese HELIUS participants, Amsterdam, the Netherlands, April 29, 2020 to September 6, 2021.

**Table S7**. Univariable and multivariable models with potential determinants of SARS-CoV-2 testing uptake among Ghanaian HELIUS participants, Amsterdam, the Netherlands, April 29, 2020 to September 6, 2021.

**Table S8**. Univariable and multivariable models with potential determinants of SARS-CoV-2 testing uptake among Turkish HELIUS participants, Amsterdam, the Netherlands, April 29, 2020 to September 6, 2021.

**Table S9**. Univariable and multivariable models with potential determinants of SARS-CoV-2 testing uptake among Moroccan HELIUS participants, Amsterdam, the Netherlands, April 29, 2020 to September 6, 2021.

**Table S10**. Test for interactions between identified determinants of lower SARS-CoV-2 uptake and ethnicity, Amsterdam, the Netherlands, April 29, 2020 to September 6, 2021.

**Methods S1. Linkage of HELIUS data with SARS-CoV-2 testing and vaccination registry data.**

In the context of infectious disease control, the Public Health Service of Amsterdam (GGD Amsterdam) collected registry data on testing and vaccination against SARS-CoV-2 for the metropolitan region of Amsterdam. These data were stored in a central database named CoronIT. HELIUS data were linked to these testing and vaccination data, while including registries dating from April 29, 2020 until September 6, 2021, using a deterministic linkage algorithm based on last name, initials, sex, date of birth, and postal code. Data protection and pseudonymization were prioritized throughout the linkage process, and was supported by a trusted third party (ZorgTTP, Houten, the Netherlands). ZorgTTP developed a secure pseudonymization software, which facilitated pseudonymization of the data at the source (i.e., at Amsterdam UMC for HELIUS and at GGD Amsterdam for CoronIT). Subsequently, the HELIUS and CoronIT data could be linked without using the original identifying variables. Only HELIUS participants who had given permission for linkage to registries and were alive on March 1, 2020 (i.e., the start of the SARS-CoV-2 pandemic) were eligible for linkage (n=20,348). Of those, 71.7% (n=14,589) were successfully linked to CoronIT data.

**Methods S2. Phases of SARS-CoV-2 testing policy in the Netherlands.**

Initially, testing for SARS-CoV-2 in the Netherlands was limited to severely ill individuals (i.e., February 27 until March 30, 2020) and between March 31 and May 31, 2020, testing was expanded to individuals with occupations needing essential contact (e.g., healthcare workers) and groups at high risk of severe COVID-19 due to medical conditions (e.g., age ≥70 years and those with comorbidities that made them eligible for annual influenza vaccination (1-3)). This first period is further referred to as phase 1. Between June 1 and November 30, 2020, testing became additionally available to anyone with COVID-19-related symptoms (phase 2). Between December 1, 2020 and March 22, 2021, testing was expanded to contacts of individuals who tested positive for SARS-CoV-2 (phase 3). From March 23, 2021 onwards, SARS-CoV-2 antigen self-tests for home use became additionally available (3, 4) (phase 4).

**Methods S3**. Interaction analyses between ethnicity and determinants of SARS-CoV-2 testing uptake.

To confirm whether certain factors were ethnic-specific, we constructed models in the total study sample, and included ethnic groups, all determinants that remained in at least one of the final multivariable models across ethnic groups, and the interaction between ethnic group and each of these determinants, one-by-one, in separate multivariable models. These models included all ethnic groups for which data were available. We tested the ethnic group and determinant interaction term using a Wald χ^2^ test.

**Results S1. Variation in PCR testing uptake over time.**

As shown in Supplementary Figure S2, PCR testing uptake at the PHS fluctuated over time. For most ethnic groups, the uptake was highest during the second (i.e., when testing was available to all individuals with COVID-19 symptoms) or third test policy phases (i.e., when testing became additionally available for anyone exposed to individuals who tested positive). However, in the African Surinamese and Ghanaian groups, uptake was highest in fourth phase (i.e., when at-home SARS-CoV-2 antigen self-tests became available).

**Results S2. Results of the interaction analyses between ethnicity and determinants of SARS-CoV-2 testing uptake.**

When testing the ethnicity and determinant interactions, we found that younger age, having attended higher vocational or university education, and higher health literacy were significantly associated with SARS-CoV-2 testing uptake in the Moroccan but not in the Dutch origin group (Supplementary Table S10). Household size was not associated with testing uptake in the Ghanaian group, whereas individuals in four-person households were more likely to test in the Dutch group. The association between overweight or obesity and testing uptake was observed in the South-Asian Surinamese but not in the Dutch group. Social support was not associated with testing uptake in the African Surinamese group, whereas limited compared to sufficient social support was associated with a lower likelihood of testing in the Dutch group.

**HELIUS baseline participants, randomly selected from the municipality registry, included between 2011 and 2015**

n=24,781

**Eligible for linkage with SARS-CoV-2 testing and vaccination registry data of the Public Health Service of Amsterdam**

n=20,348 (82.1%)

**Excluded (n=4,433)**

- No informed consent for data linkage to health registries (n=4,180)

- Deceased before March 1, 2020 (n=253)

**Participated in both physical examination and questionnaire at HELIUS baseline visit**

n=19,501 (95.8%)

**Excluded (n=847)**

- Participants who did not complete both HELIUS baseline physical examination and questionnaire

**Excluded (n=495)**

- Javanese Surinamese (n=217), other Surinamese (n=235) or unknown (n=43) ethnic origin

**Included in analyses**

n=19,006 (97.5%)

**Figure S1. Flowchart of the inclusion of HELIUS participants, Amsterdam, the Netherlands.** Abbreviations: *HELIUS* Healthy Life in an Urban Setting; *SARS-CoV-2* Severe Acute Respiratory Syndrome Coronavirus 2.

**Figure S2. Percentage of SARS-CoV-2 testing uptake among HELIUS participants during different phases of the Dutch SARS-CoV-2 testing policy, Amsterdam, the Netherlands.** Phase 1: testing was allowed for individuals with occupations needing essential contact and medical groups at high risk of SARS-CoV-2 infection (April 29-May 31, 2020); phase 2: testing became additionally available for individuals with COVID-19-related symptoms (June 1-November 30, 2020); phase 3: testing became additionally available for anyone exposed to individuals who tested positive for SARS-CoV-2 (December 1, 2020-March 22, 2021) and vaccination against SARS-CoV-2 became available on January 6, 2021, initially prioritizing vulnerable groups, such as the elderly, healthcare workers and other essential services workers, and then continued with groups decreasing in age in the general population; phase 4: at-home SARS-CoV-2 antigen self-tests became additionally available (March 23-September 6, 2021). We included SARS-CoV-2 testing data registered by the Public Health service of Amsterdam between April 29, 2020 and September 6, 2021. PCR was used to detect SARS-CoV-2 and was conducted by a testing center affiliated with the Public Health Service of Amsterdam. Abbreviations: *HELIUS* Healthy Life in an Urban Setting; *PCR* Polymerase chain reaction; *SARS-CoV-2* Severe Acute Respiratory Syndrome Coronavirus 2.

**Table S1. Characteristics of HELIUS participants who were eligible for linkage with registry data, per ethnic group, Amsterdam, the Netherlands ^a^.**

| **Characteristic** | **Total**  **(n=19,006)** | **Dutch  (n=4,230)** | **South-Asian Surinamese (n=2,734)** | **African Surinamese**  **(n=3,620)** | **Ghanaian**  **(n=2,085)** | **Turkish**  **(n=3,075)** | **Moroccan**  **(n=3,262)** |  |
| --- | --- | --- | --- | --- | --- | --- | --- | --- |
|  | n (%) | n (%) | n (%) | n (%) | n (%) | n (%) | n (%) |  |
| **Age in years**, median (IQR) ^b^ | 53 (41-62) | 54 (40-65) | 55 (43-63) | 58 (47-64) | 54 (45-61) | 49 (38-56) | 47 (36-58) |  |
| <30 | 1,367 (7.2) | 211 (5.0) | 165 (6.0) | 148 (4.1) | 126 (6.0) | 339 (11.0) | 378 (11.6) |  |
| 30-39 | 3,124 (16.4) | 810 (19.1) | 432 (15.8) | 416 (11.5) | 204 (9.8) | 587 (19.1) | 675 (20.7) |  |
| 40-49 | 3,612 (19.0) | 731 (17.3) | 431 (15.8) | 522 (14.4) | 442 (21.2) | 718 (23.3) | 768 (23.5) |  |
| 50-59 | 5,242 (27.6) | 918 (21.7) | 769 (28.1) | 1,021 (28.2) | 751 (36.0) | 964 (31.3) | 819 (25.1) |  |
| 60-69 | 4,251 (22.4) | 980 (23.2) | 691 (25.3) | 1,169 (32.3) | 521 (25.0) | 392 (12.7) | 498 (15.3) |  |
| 70-79 | 1,410 (7.4) | 580 (13.7) | 246 (9.0) | 344 (9.5) | 41 (2.0) | 75 (2.4) | 124 (3.8) |  |
| **Sex** |  |  |  |  |  |  |  |  |
| Male | 8,184 (43.1) | 1,966 (46.5) | 1,230 (45.0) | 1,436 (39.7) | 816 (39.1) | 1,420 (46.2) | 1,316 (40.3) |  |
| Female | 10,822 (56.9) | 2,264 (53.5) | 1,504 (55.0) | 2,184 (60.3) | 1,269 (60.9) | 1,655 (53.8) | 1,946 (59.7) |  |
| **Higher vocational/university education** |  |  |  |  |  |  |  |  |
| No ^c^ | 13,846 (73.5) | 1,673 (39.7) | 2,121 (78.0) | 2,793 (77.9) | 1,921 (93.7) | 2,614 (85.8) | 2,724 (84.3) |  |
| Yes | 5,000 (26.5) | 2,539 (60.3) | 598 (22.0) | 794 (22.1) | 129 (6.3) | 431 (14.2) | 509 (15.7) |  |
| **Number of people in household** |  |  |  |  |  |  |  |  |
| 1 (lives alone) | 3,878 (20.7) | 1,148 (27.2) | 621 (23.0) | 1,161 (32.6) | 309 (15.2) | 308 (10.1) | 331 (10.3) |  |
| 2 | 4,637 (24.7) | 1,716 (40.7) | 659 (24.4) | 902 (25.4) | 432 (21.3) | 494 (16.3) | 434 (13.5) |  |
| 3 | 3,513 (18.7) | 646 (15.3) | 584 (21.6) | 685 (19.3) | 487 (24.0) | 617 (20.3) | 494 (15.3) |  |
| 4 | 3,574 (19.0) | 553 (13.1) | 522 (19.3) | 521 (14.7) | 468 (23.0) | 849 (27.9) | 661 (20.5) |  |
| ≥5 | 3,160 (16.8) | 153 (3.6) | 315 (11.7) | 287 (8.1) | 335 (16.5) | 770 (25.3) | 1,300 (40.4) |  |
| *Missing* | *244* | *14* | *33* | *64* | *54* | *37* | *42* |  |
| **Diabetes mellitus** ^d^ |  |  |  |  |  |  |  |  |
| No | 16,833 (89.0) | 4,061 (96.4) | 2,185 (80.2) | 3,158 (87.9) | 1,826 (88.1) | 2,734 (89.5) | 2,869 (88.2) |  |
| Yes | 2,076 (11.0) | 151 (3.6) | 541 (19.8) | 433 (12.1) | 246 (11.9) | 322 (10.5) | 383 (11.8) |  |
| *Missing* | *97* | *18* | *8* | *29* | *13* | *19* | *10* |  |
| **Cardiovascular disease** ^e^ |  |  |  |  |  |  |  |  |
| No | 16,819 (89.0) | 4,047 (95.8) | 2,321 (85.3) | 3,218 (89.3) | 1,873 (91.2) | 2,554 (83.8) | 2,806 (86.4) |  |
| Yes | 2,082 (11.0) | 179 (4.2) | 399 (14.7) | 387 (10.7) | 180 (8.8) | 495 (16.2) | 442 (13.6) |  |
| *Missing* | *105* | *4* | *14* | *15* | *32* | *26* | *14* |  |
| **Asthma/COPD** ^f^ |  |  |  |  |  |  |  |  |
| No | 17,964 (94.5) | 4,006 (94.7) | 2,535 (92.7) | 3,443 (95.1) | 2,031 (97.4) | 2,857 (92.9) | 3,092 (94.8) |  |
| Yes | 1,042 (5.5) | 224 (5.3) | 199 (7.3) | 177 (4.9) | 54 (2.6) | 218 (7.1) | 170 (5.2) |  |
| **BMI (in kg/m^2^)** ^g^, median [IQR] | 26.4 (23.4-30.0) | 24.1 (21.9-26.8) | 25.8 (23.2-28.9) | 27.1 (24.0-30.9) | 27.9 (25.0-31.1) | 28.0 (24.7-31.7) | 27.1 (24.0-30.9) |  |
| Underweight/healthy weight (BMI <25 kg/m^2^) | 7,301 (38.4) | 2,525 (59.7) | 1,165 (42.6) | 1,189 (32.9) | 522 (25.1) | 823 (26.8) | 1,077 (33.0) |  |
| Overweight (BMI 25-30 kg/m^2^) | 6,875 (36.2) | 1,272 (30.1) | 1,040 (38.1) | 1,339 (37.1) | 856 (41.1) | 1,151 (37.5) | 1,217 (37.3) |  |
| Obesity (BMI ≥30 kg/m^2^) | 4,813 (25.3) | 430 (10.2) | 527 (19.3) | 1,085 (30.0) | 705 (33.8) | 1,099 (35.8) | 967 (29.7) |  |
| *Missing* | *17* | *3* | *2* | *7* | *2* | *2* | *1* |  |
| **Health literacy** ^h^ |  |  |  |  |  |  |  |  |
| Adequate | 16,138 (85.3) | 4,198 (99.3) | 2,527 (92.8) | 3,511 (97.2) | 1,336 (64.7) | 2,167 (71.0) | 2,399 (74.0) |  |
| Low | 2,786 (14.7) | 30 (0.7) | 196 (7.2) | 101 (2.8) | 728 (35.3) | 887 (29.0) | 844 (26.0) |  |
| *Missing* | *82* | *2* | *11* | *8* | *21* | *21* | *19* |  |
| **Experienced stress at work or at home** ^i^ |  |  |  |  |  |  |  |  |
| Never | 6,277 (33.3) | 1,030 (24.4) | 855 (31.5) | 1,349 (37.5) | 984 (48.1) | 944 (31.1) | 1,115 (34.7) |  |
| Ever | 12,555 (66.7) | 3,197 (75.6) | 1,860 (68.5) | 2,248 (62.5) | 1,061 (51.9) | 2,089 (68.9) | 2,100 (65.3) |  |
| *Missing* | *174* | *3* | *19* | *23* | *40* | *42* | *47* |  |
| **Social support** ^j^ |  |  |  |  |  |  |  |  |
| Sufficient | 12,151 (65.1) | 3,250 (77.1) | 1,702 (63.0) | 2,449 (68.6) | 1,379 (68.5) | 1,433 (47.7) | 1,938 (61.1) |  |
| Limited | 6,526 (34.9) | 968 (22.9) | 1,001 (37.0) | 1,119 (31.4) | 634 (31.5) | 1,572 (52.3) | 1,232 (38.9) |  |
| *Missing* | *329* | *12* | *31* | *52* | *72* | *70* | *92* |  |
| **Perceived discrimination because of their background** ^k^ |  |  |  |  |  |  |  |  |
| Never | 5,737 (30.5) | 3,291 (77.9) | 435 (16.0) | 405 (11.3) | 487 (23.8) | 585 (19.3) | 534 (16.5) |  |
| Ever | 13,099 (69.5) | 931 (22.1) | 2,277 (84.0) | 3,181 (88.7) | 1,563 (76.2) | 2,445 (80.7) | 2,702 (83.5) |  |
| *Missing* | *170* | *8* | *22* | *34* | *35* | *45* | *26* |  |
| **Cultural orientation** ^l^ |  |  |  |  |  |  |  |  |
| More integrated | 15,995 (85.4) | NA | 2,330 (86.3) | 3,145 (88.0) | 1,539 (75.5) | 2,138 (71.3) | 2,613 (81.8) |  |
| Less integrated | 2,741 (14.6) | NA | 371 (13.7) | 430 (12.0) | 499 (24.5) | 860 (28.7) | 581 (18.2) |  |
| *Missing* | *270* | *NA* | *33* | *45* | *47* | *77* | *68* |  |
| **Difficulty with Dutch language** ^m^ |  |  |  |  |  |  |  |  |
| No | 12,699 (67.2) | NA | 2,067 (75.9) | 3,146 (87.3) | 344 (16.7) | 1,209 (39.8) | 1,703 (52.4) |  |
| Yes | 6,211 (32.8) | NA | 657 (24.1) | 459 (12.7) | 1,721 (83.3) | 1,830 (60.2) | 1,544 (47.6) |  |
| *Missing* | *96* | *NA* | *10* | *15* | *20* | *36* | *15* |  |
| **Completed at least one SARS-CoV-2 test** ^n^ |  |  |  |  |  |  |  |  |
| No | 10,431 (54.9) | 2,192 (51.8) | 1,419 (51.9) | 2,021 (55.8) | 1,587 (76.1) | 1,504 (48.9) | 1,708 (52.4) |  |
| Yes | 8,575 (45.1) | 2,038 (48.2) | 1,315 (48.1) | 1,599 (44.2) | 498 (23.9) | 1,571 (51.1) | 1,554 (47.6) |  |
| **Number of completed tests among those with at least one SARS-CoV-2 test** |  |  |  |  |  |  |  |  |
| 1 | 4,540 (52.9) | 914 (44.8) | 690 (52.5) | 906 (56.7) | 354 (71.1) | 826 (52.6) | 850 (54.7) |  |
| 2 | 2,123 (24.8) | 491 (24.1) | 317 (24.1) | 415 (26.0) | 99 (19.9) | 420 (26.7) | 381 (24.5) |  |
| ≥3 | 1,912 (22.3) | 633 (31.1) | 308 (23.4) | 278 (17.4) | 45 (9.0) | 325 (20.7) | 323 (20.8) |  |
| **At least one positive test result among those with at least one SARS-CoV-2 test** |  |  |  |  |  |  |  |  |
| No | 5,995 (69.9) | 1,732 (85.0) | 930 (70.7) | 1,120 (70.0) | 359 (72.1) | 960 (61.1) | 894 (57.5) |  |
| Yes | 2,580 (30.1) | 306 (15.0) | 385 (29.3) | 479 (30.0) | 139 (27.9) | 611 (38.9) | 660 (42.5) |  |

^a^ Participants were eligible for linkage if they had provided informed consent for data linkage to health registries and were alive on March 1, 2020. ^b^ Recalculated to age on January 1, 2021. ^c^ No school/elementary school, lower/intermediate vocational school, lower/intermediate secondary school. ^d^ Based on a fasting glucose level of ≥7.0mmol/l, self-report and/or on medication use. ^e^ Measured using the Rose questionnaire. ^f^ Based on medication use. ^g^ Length and weight were measured twice during baseline physical examination, and body mass index (BMI) was calculated as weight (kg) / length (m)^2^. ^h^ Measured using the Set of Brief Screening Questions. ^i^ Ever experienced some, severe or permanent stress, measured using the INTERHEART questionnaire. ^j^ Measured using the Social Support Questionnaire for Transactions. ^k^ Ever perceived discrimination because of their background as measured using the Everyday Discrimination Scale. ^l^ Being classified as not integrated includes individuals classified as separated or marginalized by the Psychological Acculturation Scale. ^m^ Measured using two statements on difficulty with Dutch conversation and reading. ^n^ SARS-CoV-2 PCR testing data was registered between April 29, 2020 and September 6, 2021 and was conducted by a testing center affiliated with the Public Health Service of Amsterdam.

Abbreviations**:** *HELIUS* Healthy Life in an Urban Setting; *IQR* interquartile range; *COPD* Chronic Obstructive Pulmonary Disease*; BMI* Body mass index; *NA* Not applicable; *SARS-CoV-2* Severe Acute Respiratory Syndrome Coronavirus 2; *PCR* Polymerase chain reaction.

**Table S2. Characteristics of HELIUS participants ^a^ for those who were and were not tested for SARS-CoV-2 (April 29, 2020 to September 6, 2021), Amsterdam, the Netherlands ^b^.**

| **Variable** | **Never tested**  **(n=10,431)** | **Ever tested**  **(n=8,575)** |  |
| --- | --- | --- | --- |
|  | n(%) | n(%) | *P* value |
| **Age in years** ^c^  median (IQR) | 54.7 (42.8-63.2) | 50.8 (39.1-59.8) | <0.001 |
| <30 | 651 (6.2) | 716 (8.3) |  |
| 30-39 | 1,518 (14.6) | 1,606 (18.7) |  |
| 40-49 | 1,841 (17.6) | 1,771 (20.7) |  |
| 50-59 | 2,860 (27.4) | 2,382 (27.8) |  |
| 60-69 | 2,620 (25.1) | 1,631 (19.0) |  |
| 70-79 | 941 (9.0) | 469 (5.5) |  |
| **Sex** |  |  | <0.001 |
| Male | 4,647 (44.5) | 3,537 (41.2) |  |
| Female | 5,784 (55.5) | 5,038 (58.8) |  |
| **Higher vocational/university education** |  |  | <0.001 |
| No ^d^ | 7,931 (76.8) | 5,915 (69.4) |  |
| Yes | 2,398 (23.2) | 2,602 (30.6) |  |
| *Missing* | *102* | *58* |  |
| **Number of people in household** |  |  | <0.001 |
| 1 (lives alone) | 2,334 (22.7) | 1,544 (18.2) |  |
| 2 | 2,643 (25.7) | 1,994 (23.5) |  |
| 3 | 1,868 (18.2) | 1,645 (19.4) |  |
| 4 | 1,814 (17.7) | 1,760 (20.7) |  |
| ≥5 | 1,609 (15.7) | 1,551 (18.3) |  |
| *Missing* | *163* | *81* |  |
| **Diabetes** ^e^ |  |  | <0.001 |
| No | 9,090 (87.6) | 7,743 (90.7) |  |
| Yes | 1,283 (12.4) | 793 (9.3) |  |
| *Missing* | *58* | *39* |  |
| **Cardiovascular disease** ^f^ |  |  | 0.250 |
| No | 9,238 (89.2) | 7,581 (88.7) |  |
| Yes | 1,116 (10.8) | 966 (11.3) |  |
| *Missing* | *77* | *28* |  |
| **Asthma/****Chronic Obstructive Pulmonary Disease** ^g^ |  |  | 0.410 |
| No | 9,872 (94.6) | 8,092 (94.4) |  |
| Yes | 559 (5.4) | 483 (5.6) |  |
| *Missing* |  |  |  |
| **Body Mass Index (BMI)** ^h^ |  |  | <0.001 |
| Underweight/healthy weight (BMI <25 kg/m^2^) | 3,893 (37.4) | 3,408 (39.8) |  |
| Overweight (BMI 25-30 kg/m^2^) | 3,787 (36.3) | 3,088 (36.0) |  |
| Obesity (BMI ≥30 kg/m^2^) | 2,743 (26.3) | 2,070 (24.2) |  |
| *Missing* | *8* | *9* |  |
| **Health literacy** ^i^ |  |  | <0.001 |
| Adequate | 8,593 (82.8) | 7,545 (88.3) |  |
| Low | 1,788 (17.2) | 998 (11.7) |  |
| *Missing* | *50* | *32* |  |
| **Experienced stress at work or at home** ^j^ |  |  | <0.001 |
| Never | 3,756 (36.4) | 2,521 (29.6) |  |
| Ever | 6,568 (63.6) | 5,987 (70.4) |  |
| *Missing* | *107* | *67* |  |
| **Social support** ^k^ |  |  | 0.023 |
| Sufficient | 6,590 (64.3) | 5,561 (65.9) |  |
| Limited | 3,653 (35.7) | 2,873 (34.1) |  |
| *Missing* | *188* | *141* |  |
| **Perceived discrimination because of their background** ^l^ |  |  | 0.910 |
| Never | 3,149 (30.5) | 2,588 (30.4) |  |
| Ever | 7,178 (69.5) | 5,921 (69.6) |  |
| *Missing* | *104* | *66* |  |
| **Cultural orientation** ^m*^ |  |  | 0.057 |
| More integrated | 6,503 (80.6) | 5,262 (81.8) |  |
| Less integrated | 1,570 (19.5) | 1,171 (18.2) |  |
| *Missing* | *166* | *104* |  |
| **Difficulty with Dutch language** ^n*^ |  |  | <0.001 |
| No | 4,364 (53.3) | 4,105 (63.2) |  |
| Yes | 3,819 (46.7) | 2,392 (36.8) |  |
| *Missing* | *56* | *40* |  |

^a^ Characteristics were measured at the HELIUS baseline visit (2011-2015) (5, 6). ^b^ SARS-CoV-2 testing data was registered between April 29, 2020 and September 6, 2021 and was conducted by a testing center affiliated with the Public Health Service of Amsterdam. ^c^ Recalculated to age on January 1, 2021. ^d^ No school/elementary school, lower/intermediate vocational school, lower/intermediate secondary school. ^e^ Based on a fasting glucose level of ≥7.0mmol/l, self-report and/or on medication use. ^f^ Measured using the Rose questionnaire. ^g^ Based on medication use. ^h^ Length and weight were measured twice during baseline physical examination, and body mass index (BMI) was calculated as weight (kg) / length (m)^2^. ^i^ Measured using the Set of Brief Screening Questions. ^j^ Ever experienced some, severe or permanent stress, measured using the INTERHEART questionnaire. ^k^ Measured using the Social Support Questionnaire for Transactions. ^l^ Ever perceived discrimination because of their background as measured using the Everyday Discrimination Scale. ^m^ Being classified as not integrated includes individuals classified as separated or marginalized by the Psychological Acculturation Scale. ^n^ Measured using two statements on difficulty with Dutch conversation and reading. ^*^ Excluding the Dutch group.

Abbreviations**:** *HELIUS* Healthy Life in an Urban Setting; *PCR* Polymerase chain reaction; *SARS-CoV-2* Severe Acute Respiratory Syndrome Coronavirus 2; *IQR* Interquartile range.

**Table S3.****Ethnic variation in SARS-CoV-2 testing uptake among HELIUS participants, Amsterdam, the Netherlands, April 29, 2020 to September 6, 2021** **(n=19,006) ^a^.**

|  | **Uncorrected model** ^b^ | | **Model corrected for population structure in Amsterdam** ^bc^ | |
| --- | --- | --- | --- | --- |
|  | OR (95% CI) | *P* value | aOR (95% CI) | *P* value |
| **Ethnicity** |  | <0.001 ^*^ |  | <0.001 ^*^ |
| Dutch | Ref | Ref | Ref | Ref |
| South-Asian Surinamese | 1.00 (0.91-1.10) | 0.947 | 0.99 (0.89-1.10) | 0.885 |
| African Surinamese | 0.85 (0.78-0.93) | <0.001 | 0.88 (0.79-0.97) | 0.007 |
| Ghanaian | 0.34 (0.30-0.38) | <0.001 | 0.35 (0.30-0.40) | <0.001 |
| Turkish | 1.12 (1.02-1.23) | 0.014 | 1.12 (1.01-1.24) | 0.027 |
| Moroccan | 0.98 (0.89-1.07) | 0.643 | 0.98 (0.89-1.08) | 0.739 |

^a^ SARS-CoV-2 testing data was registered between April 29, 2020 and September 6, 2021. PCR was used to detect SARS-CoV-2 and was conducted by a testing center affiliated with the Public Health Service of Amsterdam. ^b^ The association between ethnicity and the uptake of at least one SARS-CoV-2 test was examined using logistic regression. ^c^ Models account for the age and sex distribution of the Amsterdam population through post-stratification weights. ^*^ Overall *P* value.

Abbreviations: *SARS-CoV-2* Severe Acute Respiratory Syndrome Coronavirus 2; *HELIUS* Healthy Life in an Urban Setting; *PCR* Polymerase chain reaction; *OR* odds ratio; *aOR* adjusted odds ratio; *CI* confidence interval; *Ref* Reference category.

**Table S4.** **Univariable and multivariable models with potential determinants of SARS-CoV-2 testing uptake among Dutch HELIUS participants, Amsterdam, the Netherlands, April 29, 2020 to September 6, 2021 ^a^.**

|  | **Univariable models** | | **Multivariable model (n=4,203)** | | |
| --- | --- | --- | --- | --- | --- |
| **Variable** | OR (95%CI) | *P* value | aOR (95%CI) | *P* value | |
| **Age in years** ^b^ |  | <0.001 ^*^ |  | <0.001 ^*^ |  |
| <30 | Ref | Ref | Ref | Ref |  |
| 30-39 | 0.96 (0.71-1.29) | 0.769 | 1.03 (0.76-1.41) | 0.835 |  |
| 40-49 | 0.92 (0.67-1.25) | 0.580 | 0.93 (0.68-1.28) | 0.662 |  |
| 50-59 | 0.88 (0.65-1.19) | 0.397 | 0.91 (0.67-1.24) | 0.557 |  |
| 60-69 | 0.75 (0.56-1.01) | 0.057 | 0.83 (0.61-1.13) | 0.239 |  |
| 70-79 | 0.42 (0.30-0.58) | <0.001 | 0.51 (0.37-0.72) | <0.001 |  |
| **Female sex (versus male)** | 1.14 (1.00-1.30) | 0.043 |  |  |  |
| **Higher vocational/university education (versus other educational backgrounds)** | 1.22 (1.07-1.39) | 0.003 |  |  |  |
| **Number of household members** |  | <0.001 ^*^ |  | 0.025 ^*^ |  |
| **1 (lives alone)** | Ref | Ref | Ref | Ref |  |
| **2** | 0.93 (0.80-1.10) | 0.400 | 0.92 (0.78-1.08) | 0.324 |  |
| **3** | 1.23 (1.00-1.51) | 0.050 | 1.14 (0.92-1.40) | 0.235 |  |
| **4** | 1.38 (1.12-1.72) | 0.003 | 1.28 (1.03-1.61) | 0.030 |  |
| **≥5** | 1.32 (0.92-1.88) | 0.134 | 1.21 (0.84-1.74) | 0.302 |  |
| **Diabetes** | 0.65 (0.46-0.90) | 0.011 |  |  |  |
| **Cardiovascular disease** | 1.03 (0.74-1.41) | 0.878 |  |  |  |
| **Asthma/Chronic Obstructive Pulmonary Disease** | 1.10 (0.82-1.46) | 0.529 |  |  |  |
| **Body Mass Index (BMI)** |  | 0.026 ^*^ |  |  |  |
| Underweight/healthy weight (BMI <25 kg/m^2^) | Ref | Ref |  |  |  |
| Overweight (BMI 25-30 kg/m^2^) | 0.84 (0.73-0.97) | 0.018 |  |  |  |
| Obesity (BMI ≥30 kg/m^2^) | 0.82 (0.66-1.02) | 0.075 |  |  |  |
| **Low subjective health literacy (versus adequate)** | 1.15 (0.54-2.47) | 0.719 |  |  |  |
| **Ever experienced stress at work or at home** | 1.30 (1.12-1.52) | 0.001 | 1.18 (1.01-1.39) | 0.039 |  |
| **Limited social support (versus sufficient)** | 0.74 (0.64-0.87) | <0.001 | 0.76 (0.65-0.90) | 0.001 |  |
| **Ever perceived discrimination based on their background** | 0.91 (0.78-1.07) | 0.256 |  |  |  |
| **Cultural orientation**, not integrated versus integrated ^c^ | NA | NA |  |  | |
| **Difficulty with Dutch language** | NA | NA |  |  | |

^a^ Univariable and multivariable logistic regression analyses were conducted to identify determinants of SARS-CoV-2 testing uptake. PCR was used to detect SARS-CoV-2 and was conducted by a testing center affiliated with the Public Health Service of Amsterdam. Determinants were measured at the HELIUS baseline visit (2011-2015) (5, 6). Models account for the age and sex distribution of the Amsterdam population through post-stratification weights. ^b^ Recalculated to age on January 1, 2021. ^c^ Being classified as not integrated includes individuals classified as separated or marginalized. ^*^ Overall *P* value.

Abbreviations**:** *SARS-CoV-2* Severe Acute Respiratory Syndrome Coronavirus 2; *HELIUS* Healthy Life in an Urban Setting; *PCR* Polymerase chain reaction; *OR* Odds Ratio; *aOR A*djusted Odds Ratio; *CI* Confidence interval; *Ref* Reference category; *NA* Not applicable.

**Table S5. Univariable and multivariable models with potential determinants of SARS-CoV-2 testing uptake among South-Asian Surinamese HELIUS participants, Amsterdam, the Netherlands, April 29, 2020 to September 6, 2021 ^a^.**

|  | **Univariable models** | | **Multivariable model (n=2,713)** | | |
| --- | --- | --- | --- | --- | --- |
| **Variable** | OR (95%CI) | *P* value | aOR (95%CI) | *P* value | |
| **Age in years** ^b^ |  | <0.001 ^*^ |  | <0.001 ^*^ |  |
| <30 | Ref | Ref | Ref | Ref |  |
| 30-39 | 0.64 (0.45-0.92) | 0.016 | 0.60 (0.42-0.87) | 0.006 |  |
| 40-49 | 0.76 (0.52-1.10) | 0.142 | 0.69 (0.48-1.00) | 0.048 |  |
| 50-59 | 0.71 (0.51-1.00) | 0.049 | 0.67 (0.47-0.94) | 0.020 |  |
| 60-69 | 0.57 (0.40-0.80) | 0.001 | 0.52 (0.37-0.74) | <0.001 |  |
| 70-79 | 0.44 (0.29-0.65) | <0.001 | 0.42 (0.28-0.63) | <0.001 |  |
| **Female sex (versus male)** | 1.15 (0.97-1.36) | 0.099 |  |  |  |
| **Higher vocational/university education (versus other educational backgrounds)** | 1.10 (0.90-1.35) | 0.336 |  |  |  |
| **Number of household members** |  | 0.036 ^*^ |  |  |  |
| 1 (lives alone) | Ref | Ref |  |  |  |
| 2 | 1.13 (0.89-1.45) | 0.320 |  |  |  |
| 3 | 1.29 (1.00-1.66) | 0.051 |  |  |  |
| 4 | 1.43 (1.11-1.86) | 0.006 |  |  |  |
| ≥5 | 1.43 (1.06-1.93) | 0.020 |  |  |  |
| **Diabetes** | 0.77 (0.63-0.94) | 0.012 |  |  |  |
| **Cardiovascular disease** | 0.94 (0.74-1.19) | 0.597 |  |  |  |
| **Asthma/Chronic Obstructive Pulmonary Disease** | 0.90 (0.65-1.24) | 0.522 |  |  |  |
| **Body Mass Index (BMI)** |  | 0.077 ^*^ |  | 0.013 ^*^ |  |
| Underweight/healthy weight (BMI <25 kg/m^2^) | Ref | Ref | Ref | Ref |  |
| Overweight (BMI 25-30 kg/m^2^) | 1.21 (1.01-1.46) | 0.043 | 1.30 (1.07-1.58) | 0.008 |  |
| Obesity (BMI ≥30 kg/m^2^) | 1.22 (0.97-1.54) | 0.089 | 1.31 (1.03-1.66) | 0.025 |  |
| **Low subjective health literacy (versus adequate)** | 0.86 (0.62-1.18) | 0.339 |  |  |  |
| **Ever experienced stress at work or at home** | 1.43 (1.19-1.71) | <0.001 | 1.41 (1.17-1.70) | <0.001 |  |
| **Limited social support (versus sufficient)** | 0.96 (0.80-1.14) | 0.625 |  |  |  |
| **Ever perceived discrimination based on their background** | 0.99 (0.79-1.25) | 0.962 |  |  |  |
| **Cultural orientation**, not integrated versus integrated ^c^ | 1.00 (0.78-1.27) | 0.982 |  |  | |
| **Difficulty with Dutch language** | 0.83 (0.68-1.01) | 0.063 |  |  | |

^a^ Univariable and multivariable logistic regression analyses were conducted to identify determinants of SARS-CoV-2 testing uptake. PCR was used to detect SARS-CoV-2 and was conducted by a testing center affiliated with the Public Health Service of Amsterdam. Determinants were measured at the HELIUS baseline visit (2011-2015) (5, 6). Models account for the age and sex distribution of the Amsterdam population through post-stratification weights. ^b^ Recalculated to age on January 1, 2021. ^c^ Being classified as not integrated includes individuals classified as separated or marginalized. ^*^ Overall *P* value.

Abbreviations**:** *SARS-CoV-2* Severe Acute Respiratory Syndrome Coronavirus 2; *HELIUS* Healthy Life in an Urban Setting; *PCR* Polymerase chain reaction; *OR* Odds Ratio; *aOR A*djusted Odds Ratio; *CI* Confidence interval; *Ref* Reference category.

**Table S6. Univariable and multivariable models with potential determinants of SARS-CoV-2 testing uptake among African Surinamese HELIUS participants, Amsterdam, the Netherlands, April 29, 2020 to September 6, 2021 ^a^.**

|  | **Univariable models** | | **Multivariable model (n=3,510)** | | |
| --- | --- | --- | --- | --- | --- |
| **Variable** | OR (95%CI) | *P* value | aOR (95%CI) | *P* value | |
| **Age in years** ^b^ |  | <0.001 ^*^ |  | <0.001 ^*^ |  |
| <30 | Ref | Ref | Ref | Ref |  |
| 30-39 | 1.17 (0.80-1.70) | 0.420 | 1.17 (0.80-1.71) | 0.426 |  |
| 40-49 | 1.09 (0.75-1.58) | 0.649 | 1.12 (0.77-1.64) | 0.555 |  |
| 50-59 | 1.02 (0.72-1.44) | 0.921 | 1.08 (0.76-1.53) | 0.679 |  |
| 60-69 | 0.70 (0.50-0.99) | 0.042 | 0.79 (0.55-1.13) | 0.191 |  |
| 70-79 | 0.57 (0.38-0.84) | 0.005 | 0.73 (0.49-1.10) | 0.129 |  |
| **Female sex (versus male)** | 1.30 (1.12-1.51) | 0.001 | 1.23 (1.05-1.43) | 0.011 |  |
| **Higher vocational/university education (versus other educational backgrounds)** | 1.43 (1.20-1.71) | <0.001 | 1.36 (1.14-1.64) | 0.001 |  |
| **Number of household members** |  | <0.001 ^*^ |  | 0.007 ^*^ |  |
| 1 (lives alone) | Ref | Ref | Ref | Ref |  |
| 2 | 1.24 (1.01-1.51) | 0.035 | 1.19 (0.97-1.46) | 0.094 |  |
| 3 | 1.60 (1.29-1.98) | <0.001 | 1.49 (1.20-1.85) | <0.001 |  |
| 4 | 1.44 (1.14-1.81) | 0.002 | 1.34 (1.06-1.71) | 0.017 |  |
| ≥5 | 1.43 (1.08-1.91) | 0.014 | 1.31 (0.97-1.77) | 0.078 |  |
| **Diabetes** | 0.66 (0.53-0.82) | <0.001 |  |  |  |
| **Cardiovascular disease** | 1.14 (0.90-1.44) | 0.284 |  |  |  |
| **Asthma/Chronic Obstructive Pulmonary Disease** | 1.26 (0.90-1.76) | 0.184 |  |  |  |
| **Body Mass Index (BMI)** |  | 0.261 ^*^ |  |  |  |
| Underweight/healthy weight (BMI <25 kg/m^2^) | Ref | Ref |  |  |  |
| Overweight (BMI 25-30 kg/m^2^) | 1.14 (0.96-1.36) | 0.132 |  |  |  |
| Obesity (BMI ≥30 kg/m^2^) | 1.13 (0.94-1.36) | 0.185 |  |  |  |
| **Low subjective health literacy (versus adequate)** | 0.90 (0.58-1.41) | 0.659 |  |  |  |
| **Ever experienced stress at work or at home** | 1.35 (1.16-1.58) | <0.001 | 1.23 (1.05-1.45) | 0.011 |  |
| **Limited social support (versus sufficient)** | 1.00 (0.85-1.17) | 0.994 |  |  |  |
| **Ever perceived discrimination based on their background** | 1.32 (1.04-1.67) | 0.021 |  |  |  |
| **Cultural orientation**, not integrated versus integrated ^c^ | 1.11 (0.88-1.39) | 0.374 |  |  | |
| **Difficulty with Dutch language** | 1.03 (0.83-1.29) | 0.781 |  |  | |

^a^ Univariable and multivariable logistic regression analyses were conducted to identify determinants of SARS-CoV-2 testing uptake. PCR was used to detect SARS-CoV-2 and was conducted by a testing center affiliated with the Public Health Service of Amsterdam. Determinants were measured at the HELIUS baseline visit (2011-2015) (5, 6). Models account for the age and sex distribution of the Amsterdam population through post-stratification weights ^b^ Recalculated to age on January 1, 2021. ^c^ Being classified as not integrated includes individuals classified as separated or marginalized. ^*^ Overall *P* value.

Abbreviations**:** *SARS-CoV-2* Severe Acute Respiratory Syndrome Coronavirus 2; *HELIUS* Healthy Life in an Urban Setting; *PCR* Polymerase chain reaction; *OR* Odds Ratio; *aOR A*djusted Odds Ratio; *CI* Confidence interval; *Ref* Reference category.

**Table S7. Univariable and multivariable models with potential determinants of SARS-CoV-2 testing uptake among Ghanaian HELIUS participants, Amsterdam, the Netherlands, April 29, 2020 to September 6, 2021 ^a^.**

|  | **Univariable models** | | **Multivariable model (n=2,085)** | | |
| --- | --- | --- | --- | --- | --- |
| **Variable** | OR (95%CI) | *P* value | aOR (95%CI) | *P* value | |
| **Age in years** ^b^ |  | 0.004 ^*^ |  | 0.003 ^*^ |  |
| <30 | Ref | Ref | Ref | Ref |  |
| 30-39 | 0.98 (0.59-1.63) | 0.941 | 0.97 (0.59-1.62) | 0.922 |  |
| 40-49 | 0.69 (0.43-1.11) | 0.129 | 0.68 (0.43-1.10) | 0.114 |  |
| 50-59 | 0.78 (0.50-1.20) | 0.252 | 0.77 (0.50-1.18) | 0.227 |  |
| 60-69 | 0.52 (0.33-0.82) | 0.005 | 0.51 (0.32-0.80) | 0.004 |  |
| 70-79 | 0.37 (0.14-0.97) | 0.043 | 0.37 (0.14-0.97) | 0.044 |  |
| **Female sex (versus male)** | 0.95 (0.75-1.20) | 0.649 |  |  |  |
| **Higher vocational/university education (versus other educational backgrounds)** | 0.95 (0.60-1.50) | 0.819 |  |  |  |
| **Number of household members** |  | 0.572 ^*^ |  |  |  |
| 1 (lives alone) | Ref | Ref |  |  |  |
| 2 | 1.18 (0.79-1.75) | 0.424 |  |  |  |
| 3 | 1.28 (0.87-1.89) | 0.204 |  |  |  |
| 4 | 0.99 (0.67-1.47) | 0.969 |  |  |  |
| ≥5 | 1.13 (0.75-1.71) | 0.558 |  |  |  |
| **Diabetes** | 0.81 (0.56-1.16) | 0.252 |  |  |  |
| **Cardiovascular disease** | 1.30 (0.88-1.92) | 0.182 |  |  |  |
| **Asthma/Chronic Obstructive Pulmonary Disease** | 1.82 (0.95-3.49) | 0.069 | 1.99 (1.07-3.70) | 0.029 |  |
| **Body Mass Index (BMI)** |  | 0.483 ^*^ |  |  |  |
| Underweight/healthy weight (BMI <25 kg/m^2^) | Ref | Ref |  |  |  |
| Overweight (BMI 25-30 kg/m^2^) | 0.99 (0.75-1.32) | 0.964 |  |  |  |
| Obesity (BMI ≥30 kg/m^2^) | 0.86 (0.63-1.16) | 0.311 |  |  |  |
| **Low subjective health literacy (versus adequate)** | 0.72 (0.56-0.91) | 0.008 |  |  |  |
| **Ever experienced stress at work or at home** | 1.05 (0.83-1.32) | 0.711 |  |  |  |
| **Limited social support (versus sufficient)** | 1.09 (0.84-1.41) | 0.503 |  |  |  |
| **Ever perceived discrimination based on their background** | 1.16 (0.88-1.53) | 0.302 |  |  |  |
| **Cultural orientation**, not integrated versus integrated ^c^ | 0.97 (0.74-1.28) | 0.833 |  |  | |
| **Difficulty with Dutch language** | 0.70 (0.52-0.93) | 0.015 |  |  | |

^a^ Univariable and multivariable logistic regression analyses were conducted to identify determinants of SARS-CoV-2 testing uptake. PCR was used to detect SARS-CoV-2 and was conducted by a testing center affiliated with the Public Health Service of Amsterdam. Determinants were measured at the HELIUS baseline visit (2011-2015) (5, 6). Models account for the age and sex distribution of the Amsterdam population through post-stratification weights. ^b^ Recalculated to age on January 1, 2021. ^c^ Being classified as not integrated includes individuals classified as separated or marginalized. ^*^ Overall *P* value.

Abbreviations**:** *SARS-CoV-2* Severe Acute Respiratory Syndrome Coronavirus 2; *HELIUS* Healthy Life in an Urban Setting; *PCR* Polymerase chain reaction; *OR* Odds Ratio; *aOR A*djusted Odds Ratio; *CI* Confidence interval; *Ref* Reference category.

**Table S8. Univariable and multivariable models with potential determinants of SARS-CoV-2 testing uptake among Turkish HELIUS participants, Amsterdam, the Netherlands, April 29, 2020 to September 6, 2021 ^a^.**

|  | **Univariable models** | | **Multivariable model (n=2,983)** | | |
| --- | --- | --- | --- | --- | --- |
| **Variable** | OR (95%CI) | *P* value | aOR (95%CI) | *P* value | |
| **Age in years** ^b^ |  | <0.001 ^*^ |  | <0.001 ^*^ |  |
| <30 | Ref | Ref | Ref | Ref |  |
| 30-39 | 1.03 (0.78-1.35) | 0.840 | 1.03 (0.78-1.38) | 0.803 |  |
| 40-49 | 1.04 (0.79-1.35) | 0.790 | 1.05 (0.79-1.38) | 0.748 |  |
| 50-59 | 0.89 (0.70-1.15) | 0.383 | 0.95 (0.74-1.24) | 0.727 |  |
| 60-69 | 0.44 (0.33-0.60) | <0.001 | 0.51 (0.37-0.70) | <0.001 |  |
| 70-79 | 0.39 (0.23-0.66) | <0.001 | 0.52 (0.29-0.90) | 0.020 |  |
| **Female sex (versus male)** | 1.18 (1.01-1.37) | 0.036 | 1.17 (1.00-1.37) | 0.049 |  |
| **Higher vocational/university education (versus other educational backgrounds)** | 1.29 (1.04-1.60) | 0.022 | 1.29 (1.02-1.63) | 0.032 |  |
| **Number of household members** |  | <0.001 ^*^ |  | <0.001 ^*^ |  |
| 1 (lives alone) | Ref | Ref | Ref | Ref |  |
| 2 | 1.45 (1.06-1.98) | 0.021 | 1.57 (1.13-2.18) | 0.007 |  |
| 3 | 1.85 (1.36-2.50) | <0.001 | 1.85 (1.24-2.54) | <0.001 |  |
| 4 | 2.16 (1.61-2.88) | <0.001 | 2.06 (1.52-2.80) | <0.001 |  |
| ≥5 | 2.14 (1.59-2.86) | <0.001 | 2.06 (1.51-2.81) | <0.001 |  |
| **Diabetes** | 0.80 (0.63-1.02) | 0.073 |  |  |  |
| **Cardiovascular disease** | 1.00 (0.81-1.23) | 0.998 |  |  |  |
| **Asthma/Chronic Obstructive Pulmonary Disease** | 0.99 (0.73-1.33) | 0.929 |  |  |  |
| **Body Mass Index (BMI)** |  | 0.253 ^*^ |  |  |  |
| Underweight/healthy weight (BMI <25 kg/m^2^) | Ref | Ref |  |  |  |
| Overweight (BMI 25-30 kg/m^2^) | 1.02 (0.84-1.23) | 0.837 |  |  |  |
| Obesity (BMI ≥30 kg/m^2^) | 0.88 (0.73-1.07) | 0.211 |  |  |  |
| **Low subjective health literacy (versus adequate)** | 0.71 (0.60-0.84) | <0.001 |  |  |  |
| **Ever experienced stress at work or at home** | 1.22 (1.03-1.44) | 0.018 | 1.21 (1.02-1.44) | 0.033 |  |
| **Limited social support (versus sufficient)** | 0.85 (0.72-0.99) | 0.034 |  |  |  |
| **Ever perceived discrimination based on their background** | 1.09 (0.90-1.33) | 0.370 |  |  |  |
| **Cultural orientation**, not integrated versus integrated ^c^ | 0.87 (0.73-1.03) | 0.110 |  |  | |
| **Difficulty with Dutch language** | 0.75 (0.64-0.87) | <0.001 |  |  | |

^a^ Univariable and multivariable logistic regression analyses were conducted to identify determinants of SARS-CoV-2 testing uptake. PCR was used to detect SARS-CoV-2 and was conducted by a testing center affiliated with the Public Health Service of Amsterdam. Determinants were measured at the HELIUS baseline visit (2011-2015) (5, 6). Models account for the age and sex distribution of the Amsterdam population through post-stratification weights. ^b^ Recalculated to age on January 1, 2021. ^c^ Being classified as not integrated includes individuals classified as separated or marginalized. ^*^ Overall *P* value.

Abbreviations**:** *SARS-CoV-2* Severe Acute Respiratory Syndrome Coronavirus 2; *HELIUS* Healthy Life in an Urban Setting; *PCR* Polymerase chain reaction; *OR* Odds Ratio; *aOR A*djusted Odds Ratio; *CI* Confidence interval; *Ref* Reference category.

**Table S9. Univariable and multivariable models with potential determinants of SARS-CoV-2 testing uptake among Moroccan HELIUS participants, Amsterdam, the Netherlands, April 29, 2020 to September 6, 2021 ^a^.**

|  | **Univariable models** | | **Multivariable model (n=3,224)** | | |
| --- | --- | --- | --- | --- | --- |
| **Variable** | OR (95%CI) | *P* value | aOR (95%CI) | *P* value | |
| **Age in years** ^b^ |  | <0.001 ^*^ |  | <0.001 ^*^ |  |
| <30 | Ref | Ref | Ref | Ref |  |
| 30-39 | 0.95 (0.73-1.23) | 0.675 | 0.89 (0.68-1.16) | 0.378 |  |
| 40-49 | 0.87 (0.67-1.13) | 0.292 | 0.87 (0.67-1.13) | 0.296 |  |
| 50-59 | 0.58 (0.45-0.75) | <0.001 | 0.66 (0.50-0.86) | 0.002 |  |
| 60-69 | 0.43 (0.32-0.57) | <0.001 | 0.54 (0.40-0.74) | <0.001 |  |
| 70-79 | 0.35 (0.23-0.54) | <0.001 | 0.46 (0.28-0.73) | 0.001 |  |
| **Female sex (versus male)** | 1.17 (1.01-1.36) | 0.031 | 1.21 (1.04-1.41) | 0.016 |  |
| **Higher vocational/university education (versus other educational backgrounds)** | 1.96 (1.60-2.41) | <0.001 | 1.70 (1.37-2.12) | <0.001 |  |
| **Number of household members** |  | 0.636 ^*^ |  |  |  |
| 1 (lives alone) | Ref | Ref |  |  |  |
| 2 | 1.15 (0.85-1.57) | 0.368 |  |  |  |
| 3 | 1.17 (0.87-1.58) | 0.293 |  |  |  |
| 4 | 1.24 (0.93-1.65) | 0.136 |  |  |  |
| ≥5 | 1.21 (0.93-1.57) | 0.151 |  |  |  |
| **Diabetes** | 0.66 (0.52-0.82) | <0.001 |  |  |  |
| **Cardiovascular disease** | 1.00 (0.81-1.24) | 0.989 |  |  |  |
| **Asthma/Chronic Obstructive Pulmonary Disease** | 0.86 (0.62-1.20) | 0.381 |  |  |  |
| **Body Mass Index (BMI)** |  | 0.219 ^*^ |  |  |  |
| Underweight/healthy weight (BMI <25 kg/m^2^) | Ref | Ref |  |  |  |
| Overweight (BMI 25-30 kg/m^2^) | 0.93 (0.78-1.10) | 0.386 |  |  |  |
| Obesity (BMI ≥30 kg/m^2^) | 0.85 (0.71-1.02) | 0.081 |  |  |  |
| **Low subjective health literacy (versus adequate)** | 0.53 (0.44-0.62) | <0.001 | 0.76 (0.62-0.93) | 0.008 |  |
| **Ever experienced stress at work or at home** | 1.04 (0.89-1.22) | 0.608 |  |  |  |
| **Limited social support (versus sufficient)** | 0.90 (0.77-1.05) | 0.165 |  |  |  |
| **Ever perceived discrimination based on their background** | 1.11 (0.91-1.35) | 0.310 |  |  |  |
| **Cultural orientation**, not integrated versus integrated ^c^ | 0.92 (0.76-1.12) | 0.401 |  |  | |
| **Difficulty with Dutch language** | 0.63 (0.54-0.72) | <0.001 |  |  | |

^a^ Univariable and multivariable logistic regression analyses were conducted to identify determinants of SARS-CoV-2 testing uptake. PCR was used to detect SARS-CoV-2 and was conducted by a testing center affiliated with the Public Health Service of Amsterdam. Determinants were measured at the HELIUS baseline visit (2011-2015) (5, 6). Models account for the age and sex distribution of the Amsterdam population through post-stratification weights. ^b^ Recalculated to age on January 1, 2021. ^c^ Being classified as not integrated includes individuals classified as separated or marginalized. ^*^ Overall *P* value.

Abbreviations**:** *SARS-CoV-2* Severe Acute Respiratory Syndrome Coronavirus 2; *HELIUS* Healthy Life in an Urban Setting; *PCR* Polymerase chain reaction; *OR* Odds Ratio; *aOR A*djusted Odds Ratio; *CI* Confidence interval; *Ref* Reference category.

**Table S10. Test for interactions between identified determinants of lower SARS-CoV-2 uptake and ethnicity, Amsterdam, the Netherlands, April 29, 2020 to September 6, 2021 ^a^.**

|  | **Dutch** | **South-Asian Surinamese** | **African Surinamese** | **Ghanaian** | **Turkish** | **Moroccan** |
| --- | --- | --- | --- | --- | --- | --- |
| **Variable** | *P* value | *P* value | *P* value | *P* value | *P* value | *P* value |
| **Age in years** ^b^ | Ref | 0.200 | 0.802 | 0.732 | 0.900 | **0.014** |
| **Female sex** | Ref | 0.752 | 0.250 | 0.258 | 0.361 | 0.689 |
| **Higher vocational/university education (versus other educational backgrounds)** | Ref | 0.508 | 0.134 | 0.211 | 0.633 | **<0.001** |
| **Number of household members** | Ref | 0.663 | 0.872 | **0.025** | 0.126 | 0.199 |
| **Asthma/Chronic Obstructive Pulmonary Disease** | Ref | 0.394 | 0.990 | 0.260 | 0.984 | 0.297 |
| **Body Mass Index** | Ref | **0.024** | 0.174 | 0.781 | 0.377 | 0.987 |
| **Health literacy** | Ref | 0.458 | 0.397 | 0.101 | 0.162 | **0.025** |
| **Experience of stress at work or at home** | Ref | 0.214 | 0.284 | 0.504 | 0.574 | 0.456 |
| **Social support** | Ref | 0.068 | **0.044** | 0.098 | 0.412 | 0.295 |

^a^ We tested for the interaction between ethnicity and determinants of SARS-CoV-2 testing uptake that were identified in at least one ethnic group (Supplementary Table S8). The models account for the age and sex distribution of the Amsterdam population through post-stratification weights, and the models are adjusted for all other covariates that were identified as a significant determinant in at least one ethnic group. Overall *P* values are reported. Statistically significant interactions between ethnic group and the determinants of testing uptake are presented in bold. ^b^ Recalculated to age on January 1, 2021.

Abbreviations**:** *SARS-CoV-2* Severe Acute Respiratory Syndrome Coronavirus 2; *Ref* Reference category.

**References**

1. National Institute for Public Health and the Environment (RIVM). Flu vaccine. Available from: <https://www.rivm.nl/en/flu-and-flu-vaccine/vaccine>. Accessed: 9 September 2024.

2. Government of the Netherlands. Advies Outbreak Management Team (OMT) 12 maart 2020. Available from: <https://www.rijksoverheid.nl/documenten/kamerstukken/2020/03/12/advies-outbreak-management-team>. Accessed: 9 September 2024.

3. Government of the Netherlands. Coronavirus tijdlijn. Available from: <https://www.rijksoverheid.nl/onderwerpen/coronavirus-tijdlijn>. Accessed: 9 September 2024.

4. Nederlandse Vereniging voor Medische Microbiologie (NVMM). Focusonderzoek Algemene Rekenkamer: Testen op corona. Available from: <https://www.nvmm.nl/vereniging/nieuws/focusonderzoek-algemene-rekenkamer-testen-op-corona/>. Accessed: 9 September 2024.

5. Campman SL, van Rossem G, Boyd A, Coyer L, Schinkel J, Agyemang C, et al. Intent to vaccinate against SARS-CoV-2 and its determinants across six ethnic groups living in Amsterdam, the Netherlands: A cross-sectional analysis of the HELIUS study. Vaccine. 2023;41(12):2035-45.

6. Campman SL, Boyd A, Coyer L, Schinkel J, Agyemang C, Galenkamp H, et al. SARS-CoV-2 vaccination uptake in six ethnic groups living in Amsterdam, the Netherlands: A registry-based study within the HELIUS cohort. Preventive medicine. 2024;178:107822.
